# Vru (Sub0144) controls expression of proven and putative virulence determinants and alters the ability of *Streptococcus uberis* to cause disease in dairy cattle

**DOI:** 10.1099/mic.0.055863-0

**Published:** 2012-06

**Authors:** Sharon A. Egan, Philip N. Ward, Michael Watson, Terence R. Field, James A. Leigh

**Affiliations:** 1 The School of Veterinary Medicine and Science, The University of Nottingham, Sutton Bonington Campus, Sutton Bonington, Leicestershire LE12 5RD, UK; 2 Nuffield Department of Clinical Laboratory Sciences, Oxford University, John Radcliffe Hospital, Headington, Oxfordshire OX3 9DU, UK; 3 ARK-Genomics, The Roslin Institute, University of Edinburgh, Easter Bush, Midlothian EH25 9RG, UK; 4 Institute for Animal Health, Compton, Berkshire RG20 7NN, UK

## Abstract

The regulation and control of gene expression in response to differing environmental stimuli is crucial for successful pathogen adaptation and persistence. The regulatory gene *vru* of *Streptococcus uberis* encodes a stand-alone response regulator with similarity to the Mga of group A *Streptococcus*. Mga controls expression of a number of important virulence determinants. Experimental intramammary challenge of dairy cattle with a mutant of *S. uberis* carrying an inactivating lesion in *vru* showed reduced ability to colonize the mammary gland and an inability to induce clinical signs of mastitis compared with the wild-type strain. Analysis of transcriptional differences of gene expression in the mutant, determined by microarray analysis, identified a number of coding sequences with altered expression in the absence of Vru. These consisted of known and putative virulence determinants, including Lbp (Sub0145), SclB (Sub1095), PauA (Sub1785) and *hasA* (Sub1696).

## Introduction

Improvements in milking hygiene, routine dry cow therapy and culling of persistently infected animals have significantly reduced mastitis rates caused by *Streptococcus agalactiae* and *Staphylococcus aureus* (Hillerton *et al.*, 1995); however, the same is not the case for infections caused by pathogens from environmental sources, namely *Escherichia coli* and *Streptococcus uberis*.**** *S. uberis* is an opportunistic pathogen, responsible for a significant proportion of clinical cases of mastitis in the UK, and remains one of the major causative agents of bovine mastitis worldwide (Botrel *et al.*, 2010; Bradley *et al.*, 2007; Hillerton *et al.*, 1993; Olde Riekerink *et al.*, 2008; Shum *et al.*, 2009). *S. uberis* has been isolated from a range of environmental sources, including soil, pasture, faeces and bedding materials (Bramley, 1982; Field *et al.*, 2003; Lopez-Benavides *et al.*, 2007; Zadoks *et al.*, 2005), and other bovine host sites including skin, genital tract, gastrointestinal tract and tonsils, all of which are colonized asymptomatically (Cruz Colque *et al.*, 1993; Epperson *et al.*, 1993; Kruze & Bramley, 1982).

The ability of *S. uberis* to adapt to such varied niches is likely to contribute to the organisms’ continued persistence within dairy systems. Such adaptation is likely to be influenced by regulatory genes, and the characterization of their role can be used to identify specific gene subsets of relevance to different conditions. Furthermore, interference with the activity of such regulatory elements may provide novel targets against which control strategies to prevent or eliminate bacterial infections may be mounted. *Streptococcus pyogenes* [group A *Streptococcus* (GAS)] is evolutionarily closely related to *S. uberis* (Ward *et al.*, 2009), and is specifically a human pathogen with a remarkable ability to cause a wide range of infections, ranging from pharyngitis to necrotizing fasciitis and streptococcal toxic-shock syndrome (Cunningham, 2000; Johansson *et al.*, 2010). Unlike many other prokaryotes, which co-ordinate the regulation of gene expression through alternative sigma factors, GAS favours control through transcriptional regulators, which can act as both activators and repressors, and can be categorized as either response regulators or two-component signal transduction systems (TCSs) (Kreikemeyer *et al.*, 2003; Opdyke *et al.*, 2001). Analysis of GAS genomes has revealed around 13 TCSs, many of which contribute to bacterial virulence. These contain a sensor kinase that responds to changes in signal by phosphorylation of a linked DNA-binding response regulator to alter patterns of gene expression (Hondorp & McIver, 2007). GAS also frequently use so called ‘stand-alone’ response regulators that have been shown to control multiple virulence genes. The positive transcriptional regulator Mga (Caparon & Scott, 1987) activates expression of a number of GAS virulence-related genes, including M protein (*emm*) (Perez-Casal *et al.*, 1991), fibronectin-binding protein (*fba*) (Terao *et al.*, 2001), collagen-like protein (*sclA*) (Rasmussen *et al.*, 2000), a C5a peptidase (*scpA*) (Chen *et al.*, 1993) and M-like immunoglobulin-binding proteins (including *mrp* and *enn*) (Podbielski *et al.*, 1995, 1996).

Coding sequences (CDSs) homologous to the regulator Mga and some of the regulated CDSs (in particular M protein and C5a peptidase) have been identified in other streptococci of human and/or animal origin, including *Streptococcus pneumoniae*, *Streptococcus dysgalactiae* and *Streptococcus iniae* (Locke *et al.*, 2008; Tettelin *et al.*, 2001; Vasi *et al.*, 2000). As such, these sequences can be postulated to represent a conserved regulon of products linked to early colonization and infection in a range of streptococcal species.

Analysis of the *S. uberis* genome has identified a number of potential regulatory elements, one of which, s*ub0144* (*vru*); shows some similarity to Mga of *S. pyogenes*. The translation product of the putative transcriptional regulator (Vru) displays 38 % sequence identity (57 % similarity) to Mgc, a transcriptional regulator common to group C streptococci (GCS) (Geyer & Schmidt, 2000), and 33 % identity (56 % similarity) to Mga, the counterpart in GAS. The *S. uberis* 0140J Vru CDS displays good homology to functional regions of these regulators extending over the fourth crucial helix–turn–helix DNA-binding domain identified in GAS Mga (McIver & Myles, 2002), supporting a putative DNA-binding mode of action in *S. uberis*. However unlike GAS and GCS, *S. uberis* lacks many of virulence determinants typically controlled via Mga/Mgc (Ward *et al.*, 2009), although CDSs with limited similarity to both C5a peptidase (*sub1154*) and collagen-like protein (*sub1095*) have been identified (Ward *et al.*, 2009). Both of these sequences, along with that of *sub0145*, shown previously to encode a lactoferrin-binding protein, have been demonstrated to play a role in virulence of *S. uberis*; in each case the absence of the gene product reduces the ability to colonize and/or cause disease in the target species (Leigh *et al.*, 2010).

In this study, we investigated the role of Vru *in vivo* and the effect of its absence on transcriptional control of gene expression using a custom-designed genome-wide microarray.

## Methods

### 

#### Bacterial strains and media.

*S. uberis* strain 0140J (strain ATCC BAA-854/0140J), originally isolated from a clinical case of bovine mastitis in the UK, was used throughout this study. The *sub0145*, *sub0826*, *sub0888* and *sub1095* mutants were isolated following PCR screening of an *S. uberis* 0140J/pGh9:*ISS1* mutant bank, as previously described (Egan *et al.*, 2010; Leigh *et al.*, 2010; Ward *et al.*, 2001). The *vru* mutant, containing an insertional disruption in the reverse orientation to the CDS, was isolated from the same mutant bank using a phenotypic screening method by identification of zones of clearing on Todd–Hewitt agar containing skimmed milk (1 %, v/v) and bovine plasminogen, as previously described (Ward *et al.*, 2003). Southern analysis and DNA sequencing were used to confirm the nature and location of mutation events (data not shown). Strains were routinely grown in Todd–Hewitt broth (THB) (Oxoid) and plated on sheep blood agar containing 1 % (w/v) aesculin (ABA) at 37 °C.

#### Isolation of bacterial RNA.

Aliquots of bacterial culture (15–20 ml) of *S. uberis* 0140J and *vru* mutant were harvested once cultures reached OD_550_ 0.42 for early exponential or OD_550_ 0.75 for late-exponential growth phase. Two volumes of RNAprotect Bacteria Reagent (Qiagen) were immediately added to each sample and the mixture was incubated for 5 min at room temperature. Samples were centrifuged (5000 ***g***, 10 min), the supernatant was discarded and the cell pellets were stored at −80 °C until RNA extraction was performed.

Total RNA was extracted following resuspension of the cells in 1 ml RLT buffer (Qiagen) containing 1 % (v/v) β-mercaptoethanol (Sigma–Aldrich). Cells were mechanically disrupted in a Mini-Beadbeater-8 (Biospec) using acid-washed glass beads (Sigma). Following disruption, glass beads and cell debris were removed by centrifugation (17 000 ***g***, 10 s). Supernatants were removed and mixed with an equal volume of 70 % ethanol. Total RNA was extracted using an RNeasy column (Qiagen) according to the manufacturer’s instructions, with RNA finally eluted in 50 µl nuclease-free water. Contaminating DNA was removed from the samples by DNase treatment using a DNA-free DNase Treatment kit (Ambion), and resulting samples were further purified by ethanol precipitation. The integrity and quality of the RNA were determined by gel electrophoresis and by spectrophotometry using an ND-1000 spectrophotometer (NanoDrop Technologies).

#### Design and hybridization to *S. uberis* 0140J gene expression microarray.

A custom-designed *S. uberis* 0140J DNA microarray was generated and probed by Oxford Gene Technology (OGT). The array encompassed the whole bacterial genome and was composed of 3780 features, with optimized 60-mer oligonucleotide probes printed in triplicate. Two separate probes were designed to match each individual gene determined from the *S. uberis* 0140J genome annotation (Ward *et al.*, 2009), with regions of homology discounted. Arrays were printed on glass microscope slides, as described elsewhere (Hughes *et al.*, 2001).

Fluorescently labelled probes for microarray hybridization were generated from 500 ng total RNA using the MessageAmp II-Bacteria RNA Amplification kit (Ambion) according to the manufacturer’s instructions. A total of 5 µg of the resulting amplified antisense RNA was purified using the RNeasy MinElute Cleanup kit (Qiagen) and hybridization was performed using a Gene Expression Hybridization kit (Agilent). Samples were labelled with either Cy3 or Cy5 *N*-hydroxysuccinimide ester reactive dyes (GE healthcare) according to the manufacturer’s instructions, with sample combinations generated following a loop design (Townsend, 2003). Three independent repetitions were performed of two growth-point comparisons, giving a total of six microarray hybridizations. Cy3- and Cy5-labelled probes were mixed together in the appropriate combinations, with the fragmented RNA and hybridization buffer applied to the microarray hybridization chamber (Agilent Technologies) and incubated at 65 °C for 17 h with 10 r.p.m. rotation. Following hybridization, microarrays were washed using Gene Expression Wash Buffer (Agilent Technologies). Arrays were scanned on an Agilent C scanner at 5 µm using the XDR function at 10 %. The images were then feature-extracted using Agilent feature extraction version 10.5.1.1 and the GE2_105_Jan09 extraction protocol.

#### Bioinformatic and statistical analyses.

The microarray data were loess- and scale-normalized (Smyth & Speed, 2003), and analysed for differential expression using limma (Smyth, 2004). Linear models were fitted to the data to compare the *vru* mutant with the wild-type 0140J strain at both the early and late exponential growth phase. The resultant *P* values were adjusted for the false discovery rate (Benjamini & Hochberg, 1995). Expression values were visualized using ProGenExpress (Watson, 2005). Genes were linked to Kyoto Encyclopedia of Genes and Genomes (KEGG) pathways (Kanehisa & Goto, 2000) and statistically enriched pathways identified using corna (Wu & Watson, 2009). Sequence analysis of the intergenic and promoter regions of the genes *sub0144* (*vru*) and *sub0145* (*lbp*) was performed using Artemis (Rutherford *et al.*, 2000) and bprom from Softberry sequence analysis tools (http://linux1.softberry.com/berry.phtml?topic=bprom&group=programs&subgroup=gfindb).

#### Challenge of lactating dairy cows with *S. uberis* and analysis of milk samples.

The requirement of a functional *vru* gene for *S. uberis* strain 0140J virulence was determined by intramammary challenge of Holstein–Friesian cows, 2–10 weeks into their first lactation, with either *S. uberis* 0140J or Vru mutant. Animal selection was based on previously well-established criteria (Leigh *et al.*, 2010) with the same virulent wild-type strain 0140J previously used in a number of challenge experiments (Field *et al.*, 2003; Leigh *et al.*, 2010; Smith *et al.*, 2003). Bacteria were grown overnight in THB at 37 °C, and cells were recovered by centrifugation (10 000 ***g***, 10 min) and resuspended in pyrogen-free saline (Sigma). Cell suspensions were diluted to approximately 1000 c.f.u. ml^−1^ and held on ice, and the number of viable bacteria was determined from aliquots prior to and post challenge. Animals were challenged in mammary quarters by infusion of 1 ml of the suspension; one animal was challenged in two mammary quarters with 1×10^3^ c.f.u. of strain 0140J, and two animals were challenged each in two mammary quarters with a similar dose of the Vru mutant.

Following challenge, animals were milked and inspected twice daily (07 : 00 h and 15 : 30 h) and were treated with appropriate antibiotics once clinical end points had been reached using criteria previously described (Field *et al.*, 2003; Leigh *et al.*, 2010), including the presence of clotted/discoloured milk and a swollen udder quarter. Milk samples were taken at each milking and analysed for the presence of bacteria and somatic cells. The number of viable bacteria present was estimated by direct plating of up to 1 ml of each milk sample onto ABA. Samples were also diluted in saline, and 50 µl of each dilution plated directly onto ABA. In each case, the presence and/or number of *S. uberis* was determined, and the genotype of the recovered isolates was determined by RFLP analysis (Hill & Leigh, 1989) and PCR amplification of the *vru* locus. The number of somatic cells present in milk samples was determined using a DeLaval portable cell counter in line with the manufacturer’s instructions.

#### Production of recombinant Sub0145, Sub0888 and Sub1095 and corresponding antisera.

The predicted mature CDS of Lbp/Sub0145 was amplified from genomic DNA (*S. uberis* 0140J) by PCR using the primer pair 5′-GAAGATTTATTTACTATAAATAATTCAG-3′ and 5′-TAGGAAAGTTTCTGGTACCCCTTTGTTA-3′ to generate an amplicon incorporating a *Kpn*I restriction site for efficient directional cloning into a *Pvu*II, *Kpn*I and alkaline phosphatase (New England Biolabs) treated pQE1 vector (Qiagen). Similarly, the Sub1095 CDS was amplified using the primer pair 5′-CAAATGCCAAGTCATTCTCATC-3′ and 5′-CGCTTAATCACAAGGTACCTCTGA-3′ with an incorporated *Kpn*I restriction site. Amplification of the predicted mature CDS of Sub0888 was performed using the primer pair 5′-GAAGAAGTAGTGTCTTCATTAGGG-3′ and 5′-CATCTTATTTGGTCGACTACTCTTC-3′, incorporating a *Sal*I restriction site for efficient directional cloning into a *Pvu*II, *Sal*I and alkaline phosphatase-treated pQE1 vector. Ligated plasmid was generated, desalted and transformed into *E. coli* M15 pREP4 (Qiagen). Recombinant clones were selected on LB kanamycin (25 µg ml^−1^) ampicillin (50 µg ml^−1^) agar plates. Overnight cultures containing each recombinant (6×His-tagged) protein were subcultured by dilution (1 : 30) into LB broth containing 50 µg ampicillin ml^−1^ and 25 µg kanamycin ml^−1^ and grown at 37 °C with agitation (200 r.p.m.) for 2 h. Expression of the recombinant proteins, subsequent purification and generation of antisera were performed as described previously (Egan *et al.*, 2010).

#### Preparation of bacterial extracts and immunoblot analysis.

Culture supernatant samples from *S. uberis* 0140J and *vru* mutant strains grown in THB to early exponential (OD_550_ 0.42) and late-exponential (OD_550_ 0.75) growth phases were harvested following centrifugation (16 000 ***g***, 10 min). Complete Protease Inhibitor (Roche Diagnostics) was added to the supernatant, which was subsequently filter-sterilized through a 0.22 µm pore-size filter (Millipore), and proteins therein were concentrated by precipitation, as previously described (Egan *et al.*, 2010). Concentrated supernatant proteins were resuspended in SDS-loading buffer and separated by SDS-PAGE using 15 % (w/v) polyacrylamide gels. Proteins were subsequently either stained with InstantBlue (Expedeon Protein Solutions) or transferred (200 mA for 1 h in a Transblot apparatus, Bio-Rad) in transfer buffer [25 mM Tris base, 192 mM glycine, 20 % (v/v) methanol, pH 8.1–8.4] to nitrocellulose membranes for immunoblotting.

Membranes were incubated in a blocking solution [0.5 % skimmed milk (Marvel) in PBS] at 4 °C overnight, washed three times for 5 min (PBS containing 0.1 % Tween-20; PBST) and then incubated for 1 h with anti-Lbp rabbit antisera (1 : 10 000 in blocking solution) or anti-PauA EC3 (Leigh, 1994) mouse monoclonal antibody (1 : 1000 in blocking solution). Membrane washes were repeated following incubation with either goat anti-rabbit IgG conjugated to horseradish peroxidase (HRP; Southern Biotech) or rabbit anti-mouse IgG conjugated to peroxidase (Sigma), each diluted 1 : 1000 in blocking buffer, for 1 h. Membranes were washed (as above) and HRP conjugate was detected following incubation in 4-chloronaphthol (0.5 mg ml^−1^) containing methanol [16.7 % (v/v)] and H_2_O_2_ [0.00015 % (v/v)] under dark conditions for 1 h. Membranes were subsequently rinsed in PBS and allowed to dry, and were stored under light-tight conditions.

#### Detection of bacterial proteins on whole cells by ELISA.

*S. uberis* strains were grown until they reached OD_550_ 0.42 or 0.75, and cultures were centrifuged (16 000 ***g***, 5 min) and bacterial pellets washed in PBS, then recentrifuged, as before. Bacteria were resuspended in ELISA coating buffer (carbonate/bicarbonate buffer, pH 9.6; Sigma) and each suspension was adjusted to OD_550_ 1. Triplicate wells were coated with 100 µl of each bacterial strain and incubated at 4 °C overnight. Wells were washed five times with PBST, and 300 µl blocking solution [0.5 % (w/v) casein/PBS] was added and plates were incubated at 4 °C overnight. Blocked wells were washed as above and individual antisera added at the following concentrations: anti-Sub0145 1 : 4000, anti-Sub0826 (Egan *et al.*, 2010) and anti-Sub0888 1 : 2000, and anti-Sub1095 1 : 500 in blocking solution, and plates were incubated for 1 h at room temperature. After repeated washes with PBST, 100 µl anti-rabbit–HRP conjugate (Sigma) was added at a dilution of 1 : 5000 in blocking solution to each well and incubated at room temperature for 1 h. Wells were then emptied and reactions developed by adding 100 µl 3,3′,5,5′-Tetramethylbenzidine (TMB) Liquid Substrate (Sigma) and stopped by the addition of 100 µl Stop Reagent for TMB Substrate (Sigma). The colorimetric reaction was measured by *A*_450_, and differences between datasets were assessed using one-way ANOVA followed by Tukey’s multiple comparisons to determine statistically significant values between each mutant strain and test antigen.

## Results

### Comparison of bacterial virulence by experimental challenge with *S. uberis* 0140J and Vru mutant

The virulence of the *S. uberis* 0140J strain and that of the mutant with a defective virulence regulator*y* gene (*vru*, *sub0144*) was assessed using a well-established experimental model for bovine mastitis (Field *et al.*, 2003; Leigh *et al.*, 2010; Smith *et al.*, 2003). The Vru mutant contained an insertional disruption (the 8 bp repeat signature of which spanned from −5 bp upstream of the ATG start codon to +3 bp), which was confirmed by PCR and DNA sequencing (data not shown).

**Fig. 1.**
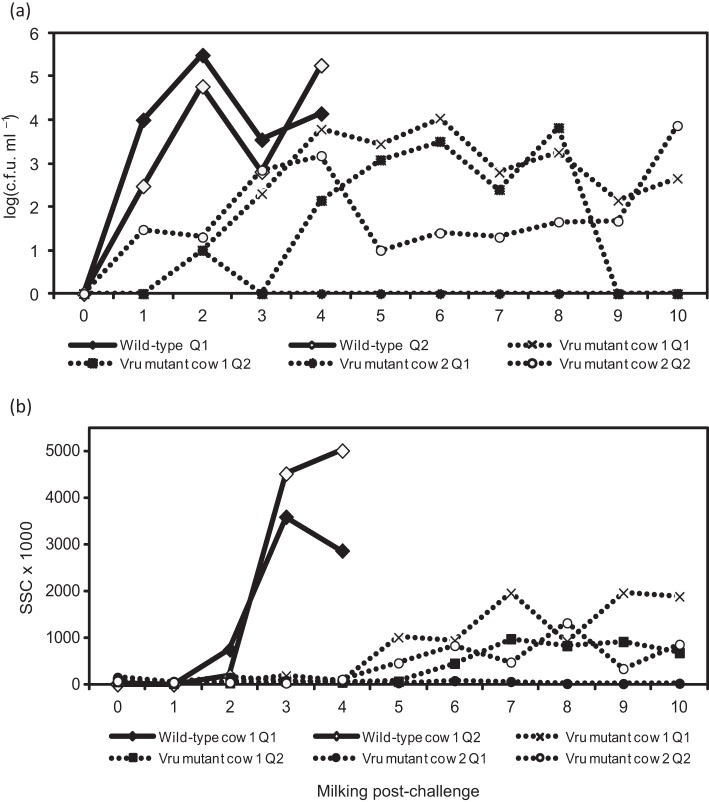
Bacterial isolation and somatic cell count following challenge with *S. uberis* 0140J and Vru mutant in dairy cattle. (a) Bacterial recovery of *S. uberis*, quantified after each milking following challenge. Data are represented as the number of bacteria obtained from the milk of animals challenged with either strain 0140J (*n* = 2) or the Vru mutant (*n* = 4) from each individual challenged quarter, as indicated above. (b) Cellular influx measured after each milking following challenge. Data are represented by the number of somatic cells (somatic cell count; SCC) obtained from the milk of animals challenged with either strain 0140J (*n* = 2) or the Vru mutant (*n* = 4) from each individual challenged quarter, as indicated above.

Both challenged quarters of the animal that received wild-type *S. uberis* became infected and shed bacteria at around 1×10^5^ c.f.u. ml^−1^ by 48 h post-challenge (Fig. 1a). Animals challenged with the Vru mutant shed considerably fewer bacteria in their milk. This low level of infection, which remained consistent and detectable for the remainder of the experiment (5 days), peaked at approximately 10^3^ c.f.u. ml^−1^ by milking 5 (Fig. 1a).

The cellular infiltration into the mammary gland in response to infection was markedly lower in quarters challenged with the Vru mutant (Fig. 1b), with no overt clinical signs of mastitis. In contrast, the animal challenged with the wild-type strain showed clinical signs of mastitis (severe changes in milk composition/colour and the inflammatory response of the mammary gland) and required antibiotic treatment after milking 4.

### Comparison of differences in gene transcription by microarray analysis

Gene expression profiles of *S. uberis* 0140J and isogenic *vru* mutant were compared by microarray analysis using a custom-designed *S. uberis* 0140J array optimized using two different probes per gene to match individual genes as determined from the genome annotation (Ward *et al.*, 2009). Two different growth time points were analysed (early and late-exponential phases of growth), and genes were considered to be differentially expressed in the wild-type and mutant if the difference between the two strains was greater than fourfold by analysis of the duplicate probes for each gene and the comparison of the datasets preferentially showed an adjusted *P* value of <0.001.

Using these criteria, a total of 19 genes were found to be expressed at lower levels by the Vru mutant, eight of which (*sub0051*, *sub0052*, *sub0145*, *sub1480*, *sub1174*, *sub1696*, *sub1697* and *sub1785*) were common to both early and late-exponential growth phases (Table 1). Genes relating to capsule production (Ward *et al.*, 2001), *hasA* and *hasB1* (*sub1697* and *sub1696*, respectively), were significantly and substantially downregulated in the Vru mutant compared with wild-type in both early (>250-fold) and late (>13-fold) exponential phases of growth. Also, both *pauA* (*sub1785*), previously shown to encode a plasminogen activator (Rosey *et al.*, 1999), and *lbp* (*sub0145*), encoding a lactoferrin-binding protein (Moshynskyy *et al.*, 2003), were dramatically downregulated in the Vru mutant cultures during early exponential growth (>100-fold) and to a substantial, but lesser, extent (>20-fold) during late growth periods. Pathway analysis showed differences in the exponential phase of growth, where two pathways were significantly downregulated in the Vru mutant compared with the wild-type 0140J strain, i.e. the valine, leucine and isoleucine biosynthesis and aminoacyl-tRNA biosynthesis pathways (Table 2).

**Table 1.** Differentially transcribed genes expressed at a higher level in early and late-exponential phase cultures of *S. uberis* 0140J compared with the *S. uberis* Vru mutant

Gene*	Protein annotation	Early exponential growth phase		Late-exponential growth phase	
		Fold change†	*P* value‡	Fold change†	*P* value‡
*hasA*/Sub1697	Hyaluronan synthase	380.0/250.2	<0.001	17.9/13.8	<0.001
*hasB1*/Sub1696	UDP-glucose 6-dehydrogenase 1	366.0/357.9	<0.001	8.5/8.1	0.006/<0.001
*lbp*/Sub0145	Lactoferrin-binding protein	255.1/153.8	<0.001	41.2/27.8	<0.001/0.1262
*pauA*/Sub1785	PauA protein precursor (streptokinase precursor)	169.6/168.3	<0.001	32.1/23.6	<0.001
Sub0051	Hypothetical protein	15.0/13.3	<0.001	16.6/10.5	<0.001/0.034
Sub0052	Membrane protein	14.8/14.5	<0.001	13.4/8.5	<0.001
Sub0177	RNA polymerase sigma factor protein	–	–	12.3/7.8	<0.001
Sub0176	Exported protein	–	–	9.4/6.6	<0.001
Sub1480	Permease	9.2/8.7	<0.001	8.1/4.1	<0.001
Sub1174	ABC transporter protein	8.3/4.2	0.033/0.002	16.4/12.9	<0.001
Sub0543	Dihydroxyacetone kinase subunit DhaK	–	–	8.7/5.2	<0.001
Sub1173	Membrane protein	–	–	8.5/6.7	0.027/<0.001
Sub0155	Basic membrane protein	8.1/7.7	<0.001	–	–
*sclB*/Sub1095	Collagen-like surface-anchored protein	6.9/6.5	0.012/<0.001	–	–
*pyrD*/Sub1264	Dihydroorotate dehydrogenase 1A	6.0/5.3	<0.001	–	–
Sub0118	Competence protein	–	–	5.6/4.5	<0.001
*pyrF*/Sub1025	Orotidine 5′-phosphate decarboxylase	5.4/5.0	<0.001	–	–
Sub0544	Dihydroxyacetone kinase family protein	–	–	4.9/4.4	<0.001
Sub0137	Lipoprotein	–	–	4.1/4.0	<0.001

* Gene and protein annotation according to the genomic sequence of *S. uberis* 0140J (Ward *et al.*, 2009).

† Mean fold change represented by *S. uberis* 0140J versus the *S. uberis vru* mutant for each of the probes tested.

‡ Where the *P* value for data from one of the two probes is not in excess of 0.001, the mean of both *P* values is provided.

**Table 2.** corna-based pathway analysis for early and late-exponential phase cultures of the *S. uberis* Vru mutant compared with *S. uberis* 0140J

Pathway regulation in Vru mutant compared with wild-type strain	Pathway	Total genes involved in pathway	Expectation	Observation	Fisher distribution *P* value
Downregulated in early exponential growth phase	Sub00290; valine, leucine and isoleucine biosynthesis	7	2	7	0.013
Downregulated in early exponential growth phase	Sub00970; aminoacyl-tRNA biosynthesis	25	7	16	0.015
Upregulated in late-exponential growth phase	Sub00230; purine metabolism	47	13	28	<0.0002
Upregulated in late-exponential growth phase	Sub00061; fatty acid biosynthesis	12	3	11	<0.0002

A number of CDSs were only differentially expressed in the early exponential phase of growth; those downregulated in the Vru mutant included *sub0155*, *sub1095*, *sub1264* and *sub1025*. Other CDSs (*sub0176*, *sub0177*, *sub0543*, *sub0544*, *sub1173*, *sub0118* and *sub0137*) were found to be downregulated in the Vru mutant only in the late-exponential phase of growth.

A total of 24 genes were found to be expressed at higher levels by the Vru mutant, 12 of which were common to both early and late-exponential growth (Table 3). A number of these genes were involved in purine biosynthesis, including *purH*, *purN*, *purF*, *purM*, *purC*, *purK*, *purE*, *purB* and *purD*. Pathway analysis unsurprisingly indicated that the Sub00230 purine metabolism pathway was upregulated in the absence of functional *vru* at both time points, but also identified that the Sub00061 fatty acid biosynthesis pathway was also significantly upregulated in the absence of Vru at the stationary growth phase (Table 2).

**Table 3.** Differentially transcribed genes expressed at a higher level in early and late-exponential phase cultures of the *S. uberis* Vru mutant compared with *S. uberis* 0140J

Gene*	Protein annotation	Early exponential growth phase		Late-exponential growth phase	
		Mean fold change†	Mean *P* value‡	Mean fold change†	Mean *P* value‡
*purH*/Sub0030	Bifunctional phosphoribosyl-aminoimidazolecarboxamide formyltransferase/IMP cyclohydrolase	91.1/90.0	<0.001	14.8/12.2	<0.001/0.065
Sub0865	Guanosine 5′-monophosphate oxidoreductase	83.1/75.9	<0.001	8.2/7.1	<0.001
*purN*/Sub0029	Phosphoribosylglycinamide formyltransferase	72.8/63.1	<0.001	10.9/9.1	<0.001
*purF*/Sub0027	Amidophosphoribosyltransferase	69.8/67.9	<0.001	31.0/29.7	<0.001
*purM*/Sub0028	Phosphoribosylaminoimidazole synthetase	67.7/65.0	<0.001	21.4/8.6	0.033/0.011
Sub0026	Phosphoribosylformylglycinamidine synthase protein	60.0/21.5	<0.001	50.7/40.8	<0.001
*purC*/Sub0025	Phosphoribosylaminoimidazole-succinocarboxamide synthase	20.0/18.0	<0.001	61.7/55.3	<0.001
*purA*/Sub0152	Adenylosuccinate synthetase	18.9/15.5	<0.001	–	–
Sub0987	Hypothetical protein	14.8/13.6	<0.001	–	–
*purK*/Sub0055	Phosphoribosylaminoimidazole carboxylase ATPase subunit	12.0/11.3	<0.001	21.2/13.2	<0.001
*purE*/Sub0054	Phosphoribosylaminoimidazole carboxylase catalytic subunit	8.2/7.6	<0.001	23.4/20.4	<0.001
*purB*/Sub0056	Adenylosuccinate lyase	7.0/6.5	<0.001	4.9/4.7	<0.001
*purD*/Sub0053	Phosphoribosylamine-glycine ligase	7.0/6.7	<0.001	18.9/18.0	<0.001
Sub0410	Membrane protein	7.0/6.3	<0.001	4.5/4.3	<0.001
Sub0411	PAP2 superfamily protein	6.1/5.9	<0.001	–	–
Sub0826	Surface-anchored subtilase family protein	–	–	6.6/6.1	<0.001
*fhs1*/Sub0942	Formate-tetrahydrofolate ligase	–	–	6.1/5.8	<0.001
*spxA*/Sub1777	Transcriptional regulator Spx	5.9/5.6	<0.001	–	–
Sub0746	Membrane protein	5.4/4.3	<0.001	–	–
Sub1260	Formate/nitrite transporter family protein	4.9/4.4	<0.001	–	–
*fabD*/Sub1497	Malonyl-CoA-acyl carrier protein transacylase	–	–	4.7/4.7	<0.001
*fabK*/Sub1498	Enoyl-ACP reductase	–	–	4.4/4.3	<0.001
Sub1233	Major facilitator superfamily protein	4.3/4.2	<0.001	–	–
*rpmB*/Sub0331	50S ribosomal protein L28	4.2/4.2	0.01/<0.001	–	–

* Gene and protein annotation according to the genomic sequence of *S. uberis* 0140J (Ward *et al.*, 2009).

† Mean fold change represented by *S. uberis* 0140J versus the *S. uberis vru* mutant for each of the probes tested.

‡ Where the *P* value for data from one of the two probes is not in excess of 0.001, the mean of both *P* values is provided.

### Detection of proteins in concentrated culture supernatants of the wild-type and Vru-deficient mutant and whole-cell ELISA

**Fig. 2.**
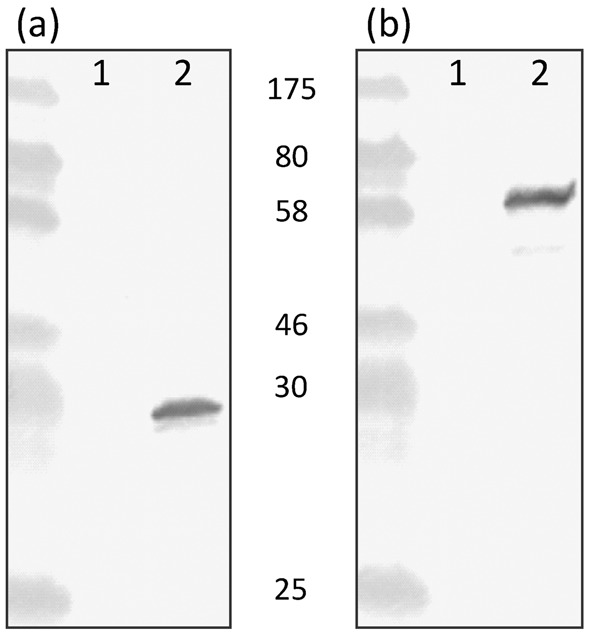
Identification of PauA and Lbp from extracts of *S. uberis* 0140J and Vru mutant. Immunoblots of protein extracts from *S. uberis* 0140J and the Vru mutant probed with either mouse monoclonal antibody generated against PauA (Sub1785) (a) or rabbit antisera generated against Lbp (Sub0145) (b). Precipitated media extracts from the late-exponential growth phase (OD_550_ 0.75) from the Vru mutant (lane 1) and 0140J (lane 2) were analysed; molecular mass standards are indicated.

Mouse monoclonal antibody directed towards PauA (Sub1785) was used to detect the presence of PauA in concentrated culture supernatants of *S. uberis* 0140J and the Vru mutant (Fig. 2a). Similarly, rabbit antisera directed against Lbp (Sub0145) were used to detect the presence of Lbp (Fig. 2b). In each case, proteins corresponding to the approximate sizes of the secreted versions of the PauA (30.6 kDa) and Lbp (54.6 kDa) proteins were detected in culture supernatant from the wild-type strain, although no cross-reactive proteins were evident in that from the Vru mutant strain.

**Fig. 3.**
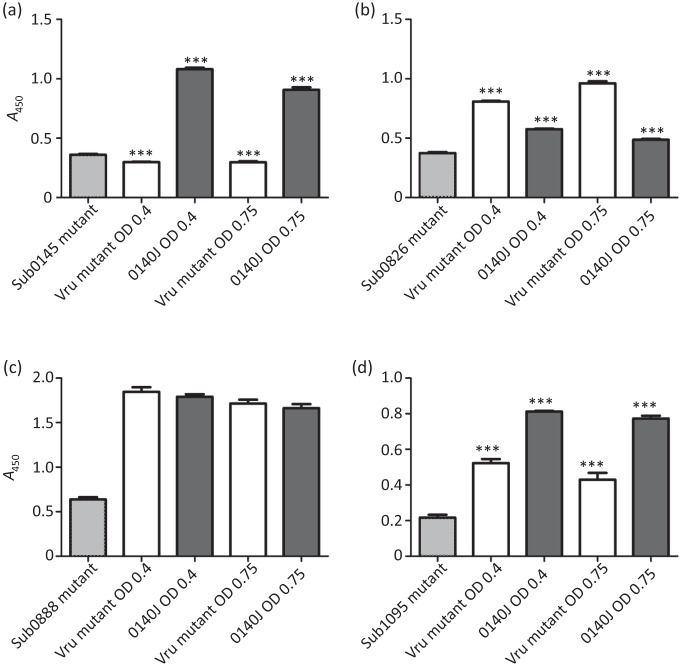
Differential protein expression between *S. uberis* 0140J and Vru mutant. Whole-cell ELISA was used to detect protein expression by *S uberis* 0140J wild-type and Vru mutant strains for Sub0145 (a), Sub0826 (b), Sub0888 (c) and Sub1095 (d). Results are expressed as the means of triplicate values; error bars, sd. In each ELISA, statistical significance was determined by ANOVA and Tukey’s multiple comparison, with triple asterisks indicating a *P* value of <0.001. The analysis displayed is between strains at same growth point, with each strain statistically significantly different from the individual mutant strain (*P*<0.001) in each assay, except in (a), in which the significant difference between the Vru mutant and Sub0145 mutant was at *P*<0.01.

As a number of the differentially expressed gene products (Sub0145, Sub0826 and Sub1095) were known to be anchored to the cell wall by SrtA in *S. uberis* (Egan *et al.*, 2010), the presence of these proteins on whole cells was determined by ELISA (Fig. 3). By way of comparison, a similarly anchored gene product (Sub0888) that appeared from the microarray analysis to be unaffected by the absence of Vru was also assayed by whole-cell ELISA. In each case the reactions of the wild-type and Vru mutant were compared with that of a relevant specific CDS mutant previously shown to lack the gene product of interest (Leigh *et al.*, 2010).

Sub0145 was only detected on the wild-type strain (Fig. 3a); the level of reaction for the Vru mutant being similar to that for the Sub0145-negative mutant. The Sub0826 protein (Fig. 3b) was detectable on both the wild-type and the Vru mutant, but at significantly higher levels on the Vru mutant compared with the wild-type. Conversely, the Sub1095 protein, which was also detected on both strains (Fig. 3d), was detected at a higher level on the wild-type than on the Vru mutant. The Sub0888 protein was detected on both strains at a similar level (Fig. 3c).

## Discussion

The ability of *S. uberis* to survive and/or proliferate within a range of external environments and in various host niches is critical for the continued persistence of this opportunistic pathogen. Such adaptation requires co-ordinated regulation of gene expression in response to differing conditions (Ward *et al.*, 2009). Survival and transmission of the pathogen between cattle may be further enhanced by the ability to control and co-ordinate virulence. In modern dairy settings, overtly infected/diseased cattle may be treated with antimicrobial compounds to eliminate the infection; however, from an evolutionary standpoint, the ability to colonize the mammary gland at high levels, coincidentally resulting in disease in the host, may favour transmission of the bacterium between individuals or niches.

The importance of the putative stand-alone gene regulator Vru for virulence of *S. uberis* was demonstrated in the current study. None of the bovine mammary quarters challenged with the Vru mutant showed high-level bacterial colonization or overt signs of mastitis; in contrast, those that received the genetically intact wild-type strain shed bacteria at high levels and displayed clinical signs similar to those previously reported following intramammary challenge with *S. uberis* (Field *et al.*, 2003; Leigh *et al.*, 2010).

The *vru* sequence exhibits a good level of homology to other stand-alone gene regulators of streptococci, *mga* and *mgc* (Caparon & Scott, 1987; Geyer & Schmidt, 2000), both of which positively influence expression of virulence-related sequences. Consistent with a role as a specific gene regulator, the absence of Vru in *S. uberis* resulted in altered levels of transcription from 42 CDSs. Of these, 19 CDSs, including five that corresponded to the production of putative virulence determinants of streptococci, were transcribed at lower levels in the absence of Vru. Conversely, 24 CDSs with higher levels of transcription were identified. It was not determined whether Vru directly or indirectly altered gene expression, but the presence of both positively and negatively altered transcription may be indicative of secondary regulatory events. To further substantiate this view, two CDSs with altered expression in the Vru mutant showed homology to other known regulatory elements. Transcription of a gene encoding an RNA polymerase sigma factor was suppressed, whereas that encoding a putative transcriptional regulator, *spxA* (Kajfasz *et al.*, 2009), was enhanced.

In the context of the current investigation it is difficult to envisage how enhanced transcription would mediate a reduction in virulence, but it was of interest to note that 10 of the 23 CDSs showing upregulation in the absence of Vru were identifiably associated with purine biosynthesis. These fell into two gene clusters: *sub0025*–*sub0030*, carrying *purCFMNH* and the unassigned CDS *sub0026*; and *sub0053*–*sub0056*, carrying *purDEKB*. A further purine biosynthesis-related sequence, *sub0152*/*purA*, located at a distant locus was also upregulated, indicating co-ordinate regulation. Lesions within purine metabolism (*purM*, *purC*) resulting in gene disruption have been shown to affect growth in raw bovine milk (Smith, 2000), but unsurprisingly, no such effect was noted for the Vru mutant.

**Fig. 4.**
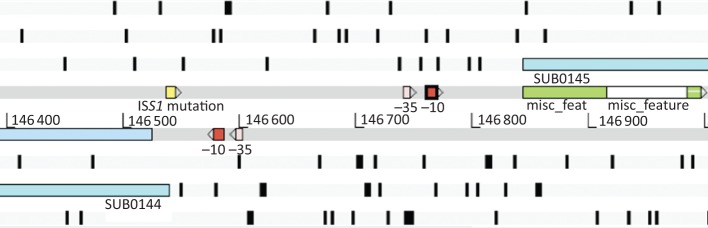
Highlighted promoter regions of the *S. uberis* 0140J, *sub0144* and *sub0145* genes. Snapshot of the Artemis program in database mode displaying the *S. uberis* 0140J chromosome, with the genes and σ^70^-type promoter regions of both *sub0144* (*vru*) and *sub0145* (*lbp*) with their orientation shown with respect to the flanking CDSs. The −10 box 5′-CATCATAAT-3′ and a −35 box, 5′-TTGAAG-3′, can be seen on the negative strand upstream of the *vru* start codon, whilst the corresponding promoter sequences are identified on the positive strand upstream of the *sub0145* gene. The location of the mutation in the *sub0144*/*vru* gene is also highlighted.

One highly downregulated sequence, *lbp*, was adjacent to *vru* within the *S. uberis* genome. We speculated whether mutation of *vru* may physically affect transcription from *lbp* by preventing read through of a single transcript. To investigate this, the 303 bp intergenic region (nucleotides 146 540–146 843 on the 0140J genome sequence) separating *sub0144*/*vru* and *sub0145* was scanned in both directions for bacterial promoter sequences using bprom and displayed using the Artemis program (Fig. 4) to illustrate promoter sequence orientation with respect to the flanking CDSs. Sequence analysis of this intergenic region identified two divergent pairs of candidate promoter sequences. This further substantiated the assertion that disruption of the *vru* ORF in the vicinity of its start codon was likely to be influencing *sub0145* expression through altered expression of its gene product, rather than abrogation of a possible shared transcriptional promoter. Furthermore, similarly altered expression of Sub0145 protein was seen in immunoblots of concentrated media proteins in a second *sub0144* mutant background where the IS*S1* insertion mapped to a position distant from candidate promoter sequences (within 100 bp of the stop codon of *sub0144*), which further excludes the possibility that both CDSs were transcribed from a shared divergent promoter (data not shown).

In GAS, the Mga regulator controls expression of a number of major virulence determinants, including M protein, C5a peptidase, ScpA, and collagen-like binding protein SclA (Barnett & Scott, 2002; Caparon & Scott, 1987; Chen *et al.*, 1993; Rasmussen *et al.*, 2000). Analysis of the *S. uberis* genome did not reveal a gene encoding M protein but did indicate a C5a peptidase-like precursor (Sub1154) and a collagen-like binding protein (Sub1095) (Egan *et al.*, 2010; Ward *et al.*, 2009). Unlike the situation with Mga in GAS, Vru did not appear to influence the expression of Sub1154, but did appear to alter expression of the collagen-like protein, which was downregulated at the transcriptional level during early exponential growth.

Hondorp & McIver (2007) speculated that only those CDSs that show high-level activation/suppression in the presence/absence of Mga (respectively) are under its direct control. A similar consideration of the data from this study would suggest that genes encoding production of capsule, plasminogen activator and lactoferrin-binding protein are those most likely to be under the direct control of Vru.

The sequences downregulated in the absence of Vru responsible for production of capsule, *hasA* and *hasB1* (Ward *et al.*, 2001), plasminogen activator, *pauA* (Leigh, 1994; Rosey *et al.*, 1999), lactoferrin-binding protein, *lbp*, and collagen-like protein, Sub1095 (Ward *et al.*, 2009), have been subject to previous investigation to determine their individual roles in virulence (Field *et al.*, 2003; Leigh *et al.*, 2010; Ward *et al.*, 2003). While Lbp and the collagen-like protein have been shown to be required for the full expression of the virulent phenotype of *S. uberis* (Leigh *et al.*, 2010), the absence of PauA or capsule alone was not deemed to affect virulence in lactating cattle (Field *et al.*, 2003; Ward *et al.*, 2003).

The transcriptional data relating to *lbp*, *pauA* and Sub1095, the collagen-like protein gene, were validated with respect to their effect on protein expression. In the absence of Vru, neither PauA nor Lbp was detectable, and the collagen-like protein, which was detected on whole cells, was shown to be present at a significantly lower level. These data, along with that related to Sub0826 (upregulated in the absence of Vru) and Sub0888 (unaffected by Vru), were consistent with their comparative transcriptional levels in the presence/absence of Vru, thus indicating that transcriptional analysis was reflected in protein expression.

It is possible that the reduced virulence observed for the Vru mutant during this study was solely due to reduced expression of Lbp. A mutant strain in the same genetic background (strain 0140J) that lacks the ability to produce Lbp has been shown to be of reduced virulence. This mutant, like the Vru mutant, is less able to colonize the lactating bovine mammary gland, and induces a lesser inflammatory response (lower somatic cell count) and fewer clinical signs than the wild type (Leigh *et al.*, 2010). However, any reduction in virulence of the Vru mutant due to the absence of Lbp may be augmented by the reduction in expression of the collagen-like protein (Sub1095), the absence of which has been shown to affect both colonization at high bacterial numbers and bacterial persistence within the bovine mammary gland (Leigh *et al.*, 2010). Although the precise mechanism by which these two proteins exert their effects on pathogenesis is currently unknown, it is unlikely that they show redundancy in their mechanisms, as the absence of each alone measurably reduced virulence. Consequently, it is likely that the absence (or reduced expression) of both has a greater effect on virulence than the absence (or reduced expression) of either alone.

A possible role for either capsule and/or the plasminogen activator PauA is more speculative. Whilst attractive as virulence determinants, neither has been shown independently to alter the virulence of *S. uberis* (Field *et al.*, 2003; Ward *et al.*, 2003), implying that either these proteins do not have a function in pathogenesis or that they exert any effect in a highly redundant manner alongside other molecules.

The hyaluronic acid capsule of a number of streptococcal species has been implicated in disease pathogenesis, linked to invasion, adherence and evasion of phagocytic host defences. Capsule production in *S. uberis* has been shown to be dependent on *hasA* and *hasC* (Ward *et al.*, 2001). This is distinct from the situation with GAS, in which capsule expression is dependent on *hasA* and *hasB* but not *hasC*. *S. uberis* carries a homologue of *hasB* (*sub1027*/*hasB2*) elsewhere within the genome (Ward *et al.*, 2009), and unlike GAS does not appear to encode another means of producing UDP-glucose, the chemical product resulting from the action of HasC. Therefore, of the genes required for capsule production in *S. uberis*, only *hasA* (as part of the *hasAB1* gene cluster) showed significantly reduced transcription in the Vru mutant; *hasC*, located around 3 kb downstream from the *hasAB1* gene cluster and *hasB2*, showed no marked differences in expression in the absence of Vru. Mutation of *hasA* in the same genetic background (strain 0140J) as used in the present study has been shown experimentally not to alter virulence. The *hasAB1* gene cluster is not universally present in *S. uberis*, although this operon has been shown to be over-represented in isolates from clinically apparent infections (Field *et al.*, 2003). Together, these data imply some level of evolutionary significance with respect to virulence; however, the precise role of the capsule, and whether it acts a genetic marker of other, more functional, CDSs remain to be determined.

Streptococcal species have evolved, or acquired by horizontal gene transfer, a number of apparently distinct products that typically activate plasminogen in a species-specific manner that invariably includes that from the target species (Caballero *et al.*, 1999; Kinnby *et al.*, 2008; Leigh *et al.*, 1998). Indeed, different strains of *S. uberis* have been shown to possess two different and mutually exclusive CDSs encoding distinct products, PauA and PauB (Rosey *et al.*, 1999; Ward & Leigh, 2002). Parallel evolution and/or acquisition and retention of such CDSs imply the utility of the activity, a view that is further reinforced by the apparent lack of variation within a selection of PauA sequences analysed (Ward & Leigh, 2004). However disabling mutations of PauA in *S. uberis* have no effect on virulence in lactating dairy cattle, indicating that any role for this activity is not linked to virulence within the lactating bovine mammary gland (Ward *et al.*, 2003). Again, the presence of PauA (or PauB) is not ubiquitous in *S. uberis*, although it appears to be present in more than 90 % of the isolates investigated (Ward & Leigh, 2002), a greater proportion than have acquired (and retained) the *hasAB1* gene cluster.

Of the remaining CDSs that were downregulated in the absence of Vru, none has previously been shown to affect virulence or have any putative or speculated role regarding the host–pathogen interaction. Some gene products were putatively involved in solute transport via ABC type transporters (Sub0137, 1174 and 1173), while others were linked to metabolism of pyrimidines (Sub1264 and 1025). However, these did not affect growth of the Vru mutant in either THB or milk.

In conclusion, it would appear justified to speculate that the observed reduced virulence of an *S. uberis* Sub0144 (Vru) mutant was mediated largely by its effect on expression from the adjacent CDS (Sub0145), encoding a surface-anchored protein shown by others to bind lactoferrin (Moshynskyy *et al.*, 2003). Such effects could be further enhanced by the concomitant reduced expression of a collagen-like protein. The apparently co-ordinated regulation of putative virulence determinants that are not directly linked with virulence, at least for the lactating bovine mammary gland, may indicate that Vru additionally or historically coordinates gene expression in *S. uberis* for attributes other than virulence following intramammary infection of the lactating bovine mammary gland.

## References

[r1] Barnett T. C. Scott J. R. **(**2002**).** Differential recognition of surface proteins in *Streptococcus pyogenes* by two sortase gene homologs. J Bacteriol 184, 2181–2191. 10.1128/JB.184.8.2181-2191.2002 11914350 PMC134975

[r2] Benjamini Y. Hochberg Y. **(**1995**).** Controlling the false discovery rate: a practical and powerful approach to multiple testing. J R Stat Soc Series B Stat Methodol 57, 289–300.

[r3] Botrel M. A. Haenni M. Morignat E. Sulpice P. Madec J. Y. Calavas D. **(**2010**).** Distribution and antimicrobial resistance of clinical and subclinical mastitis pathogens in dairy cows in Rhône-Alpes, France. Foodborne Pathog Dis 7, 479–487. 10.1089/fpd.2009.0425 19919286

[r4] Bradley A. J. Leach K. A. Breen J. E. Green L. E. Green M. J. **(**2007**).** Survey of the incidence and aetiology of mastitis on dairy farms in England and Wales. Vet Rec 160, 253–258. 10.1136/vr.160.8.253 17322356

[r5] Bramley A. J. **(**1982**).** Sources of *Streptococcus uberis* in the dairy herd. I. Isolation from bovine faeces and from straw bedding of cattle. J Dairy Res 49, 369–373. 10.1017/S0022029900022500 7142525

[r6] Caballero A. R. Lottenberg R. Johnston K. H. **(**1999**).** Cloning, expression, sequence analysis, and characterization of streptokinases secreted by porcine and equine isolates of *Streptococcus equisimilis*. Infect Immun 67, 6478–6486. 10569766 10.1128/iai.67.12.6478-6486.1999 PMC97058

[r7] Caparon M. G. Scott J. R. **(**1987**).** Identification of a gene that regulates expression of M protein, the major virulence determinant of group A streptococci. Proc Natl Acad Sci U S A 84, 8677–8681. 10.1073/pnas.84.23.8677 2446327 PMC299609

[r8] Chen C. Bormann N. Cleary P. P. **(**1993**).** VirR and Mry are homologous *trans*-acting regulators of M protein and C5a peptidase expression in group A streptococci. Mol Gen Genet 241, 685–693. 10.1007/BF00279912 7505389

[r9] Cruz Colque J. I. Devriese L. A. Haesebrouck F. **(**1993**).** Streptococci and enterococci associated with tonsils of cattle. Lett Appl Microbiol 16, 72–74. 10.1111/j.1472-765X.1993.tb00346.x 7763446

[r10] Cunningham M. W. **(**2000**).** Pathogenesis of group A streptococcal infections. Clin Microbiol Rev 13, 470–511. 10.1128/CMR.13.3.470-511.2000 10885988 PMC88944

[r11] Egan S. A. Kurian D. Ward P. N. Hunt L. Leigh J. A. **(**2010**).** Identification of sortase A (SrtA) substrates in *Streptococcus uberis*: evidence for an additional hexapeptide (LPXXXD) sorting motif. J Proteome Res 9, 1088–1095. 10.1021/pr901025w 20038184

[r12] Epperson W. B. Hoblet K. H. Smith K. L. Hogan J. S. Todhunter D. A. **(**1993**).** Association of abnormal uterine discharge with new intramammary infection in the early postpartum period in multiparous dairy cows. J Am Vet Med Assoc 202, 1461–1464. 8496101

[r13] Field T. R. Ward P. N. Pedersen L. H. Leigh J. A. **(**2003**).** The hyaluronic acid capsule of *Streptococcus uberis* is not required for the development of infection and clinical mastitis. Infect Immun 71, 132–139. 10.1128/IAI.71.1.132-139.2003 12496158 PMC143150

[r14] Geyer A. Schmidt K. H. **(**2000**).** Genetic organisation of the M protein region in human isolates of group C and G streptococci: two types of multigene regulator-like (*mgrC*) regions. Mol Gen Genet 262, 965–976. 10.1007/PL00008665 10660058

[r15] Hill A. W. Leigh J. A. **(**1989**).** DNA fingerprinting of *Streptococcus uberis*: a useful tool for epidemiology of bovine mastitis. Epidemiol Infect 103, 165–171. 10.1017/S0950268800030466 2776850 PMC2249475

[r16] Hillerton J. E. Shearn M. F. Teverson R. M. Langridge S. Booth J. M. **(**1993**).** Effect of pre-milking teat dipping on clinical mastitis on dairy farms in England. J Dairy Res 60, 31–41. 10.1017/S0022029900027321 8436665

[r17] Hillerton J. E. Bramley A. J. Staker R. T. McKinnon C. H. **(**1995**).** Patterns of intramammary infection and clinical mastitis over a 5 year period in a closely monitored herd applying mastitis control measures. J Dairy Res 62, 39–50. 10.1017/S0022029900033653 7738244

[r18] Hondorp E. R. McIver K. S. **(**2007**).** The Mga virulence regulon: infection where the grass is greener. Mol Microbiol 66, 1056–1065. 10.1111/j.1365-2958.2007.06006.x 18001346

[r19] Hughes T. R. Mao M. Jones A. R. Burchard J. Marton M. J. Shannon K. W. Lefkowitz S. M. Ziman M. Schelter J. M. **(**2001**).** Expression profiling using microarrays fabricated by an ink-jet oligonucleotide synthesizer. Nat Biotechnol 19, 342–347. 10.1038/86730 11283592

[r20] Johansson L. Thulin P. Low D. E. Norrby-Teglund A. **(**2010**).** Getting under the skin: the immunopathogenesis of *Streptococcus pyogenes* deep tissue infections. Clin Infect Dis 51, 58–65. 10.1086/653116 20491545

[r21] Kajfasz J. K. Martinez A. R. Rivera-Ramos I. Abranches J. Koo H. Quivey R. G. Jr Lemos J. A. **(**2009**).** Role of Clp proteins in expression of virulence properties of *Streptococcus mutans*. J Bacteriol 191, 2060–2068. 10.1128/JB.01609-08 19181818 PMC2655509

[r22] Kanehisa M. Goto S. **(**2000**).** KEGG: Kyoto Encyclopedia of Genes and Genomes. Nucleic Acids Res 28, 27–30. 10.1093/nar/28.1.27 10592173 PMC102409

[r23] Kinnby B. Booth N. A. Svensäter G. **(**2008**).** Plasminogen binding by oral streptococci from dental plaque and inflammatory lesions. Microbiology 154, 924–931. 10.1099/mic.0.2007/013235-0 18310038

[r24] Kreikemeyer B. McIver K. S. Podbielski A. **(**2003**).** Virulence factor regulation and regulatory networks in *Streptococcus pyogenes* and their impact on pathogen–host interactions. Trends Microbiol 11, 224–232. 10.1016/S0966-842X(03)00098-2 12781526

[r25] Kruze J. Bramley A. J. **(**1982**).** Sources of *Streptococcus uberis* in the dairy herd. II. Evidence of colonization of the bovine intestine by *Str. uberis*. J Dairy Res 49, 375–379. 10.1017/S0022029900022512 7142526

[r26] Leigh J. A. **(**1994**).** Purification of a plasminogen activator from *Streptococcus uberis*. FEMS Microbiol Lett 118, 153–158. 10.1111/j.1574-6968.1994.tb06818.x 8013872

[r27] Leigh J. A. Hodgkinson S. M. Lincoln R. A. **(**1998**).** The interaction of *Streptococcus dysgalactiae* with plasmin and plasminogen. Vet Microbiol 61, 121–135. 10.1016/S0378-1135(98)00179-5 9646471

[r28] Leigh J. A. Egan S. A. Ward P. N. Field T. R. Coffey T. J. **(**2010**).** Sortase anchored proteins of *Streptococcus uberis* play major roles in the pathogenesis of bovine mastitis in dairy cattle. Vet Res 41, 63. 10.1051/vetres/2010036 20519112 PMC2898060

[r29] Locke J. B. Aziz R. K. Vicknair M. R. Nizet V. Buchanan J. T. **(**2008**).** *Streptococcus iniae* M-like protein contributes to virulence in fish and is a target for live attenuated vaccine development. PLoS ONE 3, e2824. 10.1371/journal.pone.0002824 18665241 PMC2483786

[r30] Lopez-Benavides M. G. Williamson J. H. Pullinger G. D. Lacy-Hulbert S. J. Cursons R. T. Leigh J. A. **(**2007**).** Field observations on the variation of *Streptococcus uberis* populations in a pasture-based dairy farm. J Dairy Sci 90, 5558–5566. 10.3168/jds.2007-0194 18024747

[r31] McIver K. S. Myles R. L. **(**2002**).** Two DNA-binding domains of Mga are required for virulence gene activation in the group A streptococcus. Mol Microbiol 43, 1591–1601. 10.1046/j.1365-2958.2002.02849.x 11952907

[r32] Moshynskyy I. Jiang M. Fontaine M. C. Perez-Casal J. Babiuk L. A. Potter A. A. **(**2003**).** Characterization of a bovine lactoferrin binding protein of *Streptococcus uberis*. Microb Pathog 35, 203–215. 10.1016/S0882-4010(03)00150-5 14521879

[r33] Olde Riekerink R. G. Barkema H. W. Kelton D. F. Scholl D. T. **(**2008**).** Incidence rate of clinical mastitis on Canadian dairy farms. J Dairy Sci 91, 1366–1377. 10.3168/jds.2007-0757 18349229

[r34] Opdyke J. A. Scott J. R. Moran C. P. Jr **(**2001**).** A secondary RNA polymerase sigma factor from *Streptococcus pyogenes*. Mol Microbiol 42, 495–502. 10.1046/j.1365-2958.2001.02657.x 11703670

[r35] Perez-Casal J. Caparon M. G. Scott J. R. **(**1991**).** Mry, a *trans-*acting positive regulator of the M protein gene of *Streptococcus pyogenes* with similarity to the receptor proteins of two-component regulatory systems. J Bacteriol 173, 2617–2624. 1849511 10.1128/jb.173.8.2617-2624.1991 PMC207828

[r36] Podbielski A. Flosdorff A. Weber-Heynemann J. **(**1995**).** The group A streptococcal *virR49* gene controls expression of four structural *vir* regulon genes. Infect Immun 63, 9–20. 7806389 10.1128/iai.63.1.9-20.1995 PMC172951

[r37] Podbielski A. Woischnik M. Pohl B. Schmidt K. H. **(**1996**).** What is the size of the group A streptococcal *vir* regulon? The Mga regulator affects expression of secreted and surface virulence factors. Med Microbiol Immunol *(*Berl*)* 185, 171–181. 10.1007/s004300050028 9007823

[r38] Rasmussen M. Edén A. Björck L. **(**2000**).** SclA, a novel collagen-like surface protein of *Streptococcus pyogenes*. Infect Immun 68, 6370–6377. 10.1128/IAI.68.11.6370-6377.2000 11035747 PMC97721

[r39] Rosey E. L. Lincoln R. A. Ward P. N. Yancey R. J. Jr Leigh J. A. **(**1999**).** PauA: a novel plasminogen activator from *Streptococcus uberis*. FEMS Microbiol Lett 178, 27–33. 10.1111/j.1574-6968.1999.tb13755.x 10483719

[r40] Rutherford K. Parkhill J. Crook J. Horsnell T. Rice P. Rajandream M. A. Barrell B. **(**2000**).** Artemis: sequence visualization and annotation. Bioinformatics 16, 944–945. 11120685 10.1093/bioinformatics/16.10.944

[r41] Shum L. W. McConnel C. S. Gunn A. A. House J. K. **(**2009**).** Environmental mastitis in intensive high-producing dairy herds in New South Wales. Aust Vet J 87, 469–475. 10.1111/j.1751-0813.2009.00523.x 19930160

[r42] Smith A. J. **(**2000**).** *The identification of genes required for growth of Streptococcus uberis in milk*. PhD thesis, University of Reading, Reading, UK.

[r43] Smith A. J. Ward P. N. Field T. R. Jones C. L. Lincoln R. A. Leigh J. A. **(**2003**).** MtuA, a lipoprotein receptor antigen from *Streptococcus uberis*, is responsible for acquisition of manganese during growth in milk and is essential for infection of the lactating bovine mammary gland. Infect Immun 71, 4842–4849. 10.1128/IAI.71.9.4842-4849.2003 12933824 PMC187302

[r44] Smyth G. K. **(**2004**).** Linear models and empirical Bayes methods for assessing differential expression in microarray experiments. Stat Appl Genet Mol Biol 3, 1–25. 16646809 10.2202/1544-6115.1027

[r45] Smyth G. K. Speed T. **(**2003**).** Normalization of cDNA microarray data. Methods 31, 265–273. 10.1016/S1046-2023(03)00155-5 14597310

[r46] Terao Y. Kawabata S. Kunitomo E. Murakami J. Nakagawa I. Hamada S. **(**2001**).** Fba, a novel fibronectin-binding protein from *Streptococcus pyogenes*, promotes bacterial entry into epithelial cells, and the *fba* gene is positively transcribed under the Mga regulator. Mol Microbiol 42, 75–86. 10.1046/j.1365-2958.2001.02579.x 11679068

[r47] Tettelin H. Nelson K. E. Paulsen I. T. Eisen J. A. Read T. D. Peterson S. Heidelberg J. DeBoy R. T. Haft D. H. **(**2001**).** Complete genome sequence of a virulent isolate of *Streptococcus pneumoniae*. Science 293, 498–506. 10.1126/science.1061217 11463916

[r48] Townsend J. P. **(**2003**).** Multifactorial experimental design and the transitivity of ratios with spotted DNA microarrays. BMC Genomics 4, 41. 10.1186/1471-2164-4-41 14525623 PMC239860

[r49] Vasi J. Frykberg L. Carlsson L. E. Lindberg M. Guss B. **(**2000**).** M-like proteins of *Streptococcus dysgalactiae*. Infect Immun 68, 294–302. 10.1128/IAI.68.1.294-302.2000 10603401 PMC97134

[r50] Ward P. N. Leigh J. A. **(**2002**).** Characterization of PauB, a novel broad-spectrum plasminogen activator from *Streptococcus uberis*. J Bacteriol 184, 119–125. 10.1128/JB.184.1.119-125.2002 11741851 PMC134755

[r51] Ward P. N. Leigh J. A. **(**2004**).** Genetic analysis of *Streptococcus uberis* plasminogen activators. Indian J Med Res 119 (Suppl.), 136–140. 15232179

[r52] Ward P. N. Field T. R. Ditcham W. G. Maguin E. Leigh J. A. **(**2001**).** Identification and disruption of two discrete loci encoding hyaluronic acid capsule biosynthesis genes *hasA*, *hasB*, and *hasC* in *Streptococcus uberis*. Infect Immun 69, 392–399. 10.1128/IAI.69.1.392-399.2001 11119529 PMC97895

[r53] Ward P. N. Field T. R. Rapier C. D. Leigh J. A. **(**2003**).** The activation of bovine plasminogen by PauA is not required for virulence of *Streptococcus uberis*. Infect Immun 71, 7193–7196. 10.1128/IAI.71.12.7193-7196.2003 14638815 PMC308948

[r54] Ward P. N. Holden M. T. Leigh J. A. Lennard N. Bignell A. Barron A. Clark L. Quail M. A. Woodward J. **(**2009**).** Evidence for niche adaptation in the genome of the bovine pathogen *Streptococcus uberis*. BMC Genomics 10, 54. 10.1186/1471-2164-10-54 19175920 PMC2657157

[r55] Watson M. **(**2005**).** ProGenExpress: visualization of quantitative data on prokaryotic genomes. BMC Bioinformatics 6, 98. 10.1186/1471-2105-6-98 15829007 PMC1087476

[r56] Wu X. Watson M. **(**2009**).** corna: testing gene lists for regulation by microRNAs. Bioinformatics 25, 832–833. 10.1093/bioinformatics/btp059 19181683 PMC2654799

[r57] Zadoks R. N. Tikofsky L. L. Boor K. J. **(**2005**).** Ribotyping of *Streptococcus uberis* from a dairy’s environment, bovine feces and milk. Vet Microbiol 109, 257–265. 10.1016/j.vetmic.2005.05.008 15967600

